# Antibacterial Activity of the Essential Oil From *Litsea cubeba* Against *Cutibacterium acnes* and the Investigations of Its Potential Mechanism by Gas Chromatography-Mass Spectrometry Metabolomics

**DOI:** 10.3389/fmicb.2022.823845

**Published:** 2022-03-02

**Authors:** Jing Chen, Jianing Zhang, Longping Zhu, Chunguo Qian, Hongru Tian, Zhimin Zhao, Lu Jin, Depo Yang

**Affiliations:** ^1^School of Pharmaceutical Sciences, Sun Yat-sen University, Guangzhou, China; ^2^Guangdong Technology Research Center for Advanced Chinese Medicine, Guangzhou, China

**Keywords:** *Cutibacterium acnes*, *Litsea cubeba*, essential oil, GC-MS untargeted metabolomics, antibacterial

## Abstract

*Cutibacterium acnes* (*C. acnes*) is an anaerobic Gram-positive bacterium generally considered as a human skin commensal, but is also involved in different infections, such as *acne* and surgical infections. Although there are a variety of treatments, the side effects and the problem of bacterial drug resistance still limit their clinical usage. In this study, we found that essential oil (EO) distilled from fresh mature *Litsea cubeba* possessed promising antibacterial activity against *C. acnes.* In order to elucidate its potential mechanism, bacteriostatic activity test, Live/Dead kit assay, scanning electron microscope (SEM), transmission electron microscope (TEM), and metabolomics were employed. In addition, the content of adenosine triphosphate (ATP) in bacterium and the activities of key enzymes involved in critical metabolic pathways were detected using a variety of biochemical assays. The results showed that EO exhibited significant antibacterial activity against *C. acnes* at a minimum inhibitory concentration (MIC) of 400 μg/mL and a minimum bactericidal concentration (MBC) of 800 μg/mL, and EO could destroy *C. acnes* morphology and inhibit its growth. Moreover, results from our study showed that EO had a significant effect on the *C. acnes* normal metabolism. In total, 86 metabolites were altered, and 34 metabolic pathways related to the carbohydrate metabolism, energy metabolism, amino acid metabolism, as well as cell wall and cell membrane synthesis were perturbed after EO administration. The synthesis of ATP in bacterial cells was also severely inhibited, and the activities of key enzymes of the glycolysis and Wood-Werkman cycle were significantly affected (Pyruvate Carboxylase, Malate Dehydrogenase and Pyruvate kinase activities were decreased, and Hexokinase was increased). Taken together, these results illustrated that the bacteriostatic effect of EO against *C. acnes* by breaking the bacterial cell morphology and perturbing cell metabolism, including inhibition of key enzyme activity and ATP synthesis. The results from our study may shed new light on the discovery of novel drugs with more robust efficacy.

## Introduction

*Cutibacterium acnes* (*C. acnes*) is considered to reside in the sebaceous glands of the skin, and plays an important role in maintaining skin pH by decomposing skin triglycerides and producing fatty acids (Gribbon et al., 1993; Webster, 1995). However, it is also a conditional pathogenic bacterium that is able to cause invasive infection of organs and tissues under specific cases, such as surgery or trauma (Achermann et al., 2014; Choi et al., 2021). In addition, studies have shown that *C. acnes* is one of the main culprits of *acne* (Dreno et al., 2018). As one of the most common skin diseases with high incidence in young adulthood, *acne* can cause physical discomfort, face skin defects, or disfigurement as the long-term chronic inflammations persist. It will even cause a tremendous psychological burden to the patients, causing anxiety and even severe mental illness (Gupta and Gupta, 1998; Thomas, 2004; Park et al., 2021).

In the past, the antibiotics, such as macrolides, clindamycin, and tetracyclines, were the most common medications prescribed for *acne* (Walsh et al., 2016). Although they are still largely active against the majority of *C. acnes*, the emerging of drug resistance becomes an urgent problem (Leyden, 1976); therefore, it is not appropriate to treat *acne* with a single antibiotic. Instead, a variety of antibiotics or the combination of antibiotics and other drugs are used, but there are still many problems with these drugs in clinical use, like irritation of the skin and mucous membranes (Nyirady et al., 2001; Archer and Chang, 2004; Strauss et al., 2007; Tanghetti and Popp, 2009). In recent years, more and more attention has been paid to natural products derived from plants due to their higher efficacy and lower toxicity, which can replace or assist antimicrobial agents in inhibiting the proliferation of *C. acnes* (De Canha et al., 2019; Di Lodovico et al., 2020; Wei et al., 2021). Accumulating evidence showed that many essential oils from plants such as cloves, cactus, tea tree, and so on possessed an inhibitory effect on *C. acnes* (Fu et al., 2009; Murbach Teles Andrade et al., 2018; Ossa-Tabares et al., 2020). However, there is a lack of research on the mechanism underlying the antibacterial activity.

*Litsea cubeba* (Lour) Pers. is a deciduous shrub or small tree belonging to the genus of Litsea of the Lauraceae family, and it is an economic crop for the production of essential oil (Chen Y.C. et al., 2020). Previous studies have demonstrated that the EO has promising anti-cancer (Dalimunthe et al., 2019; Pante et al., 2021), bacteriostatic (Liu and Yang, 2012; Li et al., 2014; Nguyen et al., 2018; Dai et al., 2021) and antifungal activities (Nardoni et al., 2019), insecticidal and mosquito repellent activities (Noosidum et al., 2008; Vongsombath et al., 2012), which has been widely used in daily chemical products and food as a flavoring and preservative (Agrawal et al., 2011).

Although EO has bacteriostatic activity against many kinds of bacteria, most of them are foodborne bacteria. The inhibitory activity against *C. acnes* has not been reported. Accordingly, we investigated the activity of *Litsea cubeba* essential oil against *C. acnes* and further explored its potential mechanism by GC-MS-based non-target metabonomics for the first time. This study may provide a new perspective to understand the mechanism of inhibiting *C. acnes* in more detail.

## Materials and Methods

### Materials

*Cutibacterium acnes* (ATCC 6919, antibiotic-susceptible) strain was obtained from Guangdong Microbial Strain Preservation Center; The mediums of Brain Heart Infusion Broth and Reinforced Clostridium Aga were from HuanKai Microbial (Guangdong, China) and Shanghai Acmec Biochemical Co., Ltd, respectively; Tween 80, Anhydrous sodium sulfate, 25% Glutaraldehyde, and TERT-butyl alcohol were obtained from DAMAO; Ampicillin was from Biofrox; Glucose, Absolute ethanol, and Sodium bisulfate dihydrate were provided from Sinopharm Chemical Reagent Co., Ltd.; Sodium hydroxide was from Guangzhou Chemical Reagent Factory, Methoxyamine hydrochloride, N-methyl-N-trimethylsilyltrifluoroacetanamide (MSTFA), and Adonitol were purchased from Sigma-Aldrich; Methanol HPLC/ACS was from Energy-chemical; Hypoxic conditions were generated using an AnaeroPack (Japan Mitsubishi MGC); C7-C40 n-alkanes was purchased from Shanghai Huicheng Biotechnology Co., Ltd.

Live/Dead BacLight Bacterial Viability Kits was obtained from Thermo (Invitrogen by Thermo Fisher Scientific, L7012), Enhanced BCA Protein Assay Kit was purchased from Beyotime, Micro Malate Dehydrogenase (MDH) Assay Kit, Micro Pyruvate Carboxylase (PC) Assay Kit, and Pyruvate kinase (PK) Assay Kit were provided from Solarbio; BacTiter-GloTM Microbial Cell Viability Assay was from Promega.

### Preparation of Essential Oil From *Litsea cubeba*

The mature fruits of *Litsea cubeba* (Lour.) were collected in mid-July from Conghua District, Guangzhou City, Guangdong Province, which were identified as *Litsea cubeba* (Lour.) according to their morphological characteristics by Professor Depo Yang of the botany of the School of Pharmaceutical Sciences, Sun Yat-sen University, and the certificate specimens were kept in the herbarium of Sun Yat-sen University. The fruits that were not eaten by insects or decayed were selected and extracted by hydrodistillation for 5 h. After standing, anhydrous sodium sulfate was added to remove water, and *Litsea cubeba* essential oil (EO) was obtained. The EO was placed in a brown glass bottle, sealed, and stored at 4°C.

### Gas Chromatography-Mass Spectrometry Analysis of the Essential Oil

EO was weighed accurately and then dissolved in chromatographic grade ethyl acetate to a final concentration of 1 mg/mL, and detected by gas chromatography-mass spectrometry (GC-MS, Thermo Scientific Trace DSQ II). The GC-MS conditions were performed as previously described (Hu et al., 2019), with slight modifications (Supplementary File 1). The retention indices (RI) were calculated for all EO components by the C7-C40 n-alkanes. Based on NIST 2017 mass spectrometry library, EO constituents were identified by comparing obtained RI and data already available in the literature (Jin et al., 2015).

### Bacterial Activation and Culture

*Cutibacterium acnes* (*C. acnes*) were taken out from the ultra-low temperature refrigerator, melted and absorbed 200 μL, evenly coated on the Reinforced Clostridium Agar (RCA) plate, and cultured inverted at 37°C for 72 h under anaerobic conditions by the AnaeroPack. Then, wet sterile cotton swabs were used to scrape the *C. acnes* from the plate, and the *C. acnes* was mixed in PBS to prepare the bacterial liquid. 200 μL bacteria suspension was coated on the RCA plate and continued to culture for 72 h under anaerobic conditions at 37°C to obtain the activated bacterial cells.

### Antibacterial Activity of Essential Oil Against *Cutibacterium acnes*

#### The Minimum Inhibitory Concentration

In order to study the antibacterial activity of the EO against *C. acnes*, the minimum inhibitory concentration of bacteria was detected using the micro-broth dilution method recommended by the Clinical and Laboratory Standard Institute (CLSI, 2017). Firstly, the bacterial strain was grown to the logarithmic growth phase and diluted by the BHI medium (10^4^–10^5^ CFU/mL). Then different concentrations of EO were added to the 96-well plate, and the final drug concentration was from 0.009375 to 19.2 mg/mL. According to the Clinical and Laboratory Standards Association 2017 (CLSI, 2017), when bacteria are not visible, the administration concentration is the minimum inhibitory concentration (MIC). At the same time, the absorbance data was obtained using 96-well microplate reader at 600 nm.

#### The Minimum Bactericidal Concentration

To determine the minimum bactericidal concentration (MBC), 10 μL medium of each well with no visible bacteria growth was removed and inoculated in RCA plates. After 72 h of incubation under anaerobic conditions at 37°C, the number of surviving organisms was determined. According to the provisions of CLSI (2017) drug sensitivity test, the lowest dose concentration that can kill 99.9% of bacteria is the MBC.

#### Bacteriostatic Curve

The medium was inoculated with *C. acnes* in the logarithmic phase and the final concentration was 10^8^ CFU/mL. Then, bacteria were treated with different concentrations of EO that were dissolved with 0.1% Tween 80, respectively. And 0.1% Tween 80 was used as vehicle control. The final concentrations of EO were 4 MIC (1,600 μg/mL), 2 MIC (800 μg/mL), MIC (400 μg/mL), and 1/2 MIC (200 μg/mL). The growth of *C. acnes* in the Control and EO-treated groups was measured by monitoring the absorbance value of bacteria at 600 nm within 5 days according to literature (Kim et al., 2021).

### Live/Dead Bacterial Viability Assay

Live/Dead bacterial viability assay is a general method to determine bacterial activity according to membrane integrity (Emerson et al., 2017). Adjust the concentration of bacteria solution to 10^8^ CFU/mL. As described in section “Bacteriostatic Curve” in method, prepare different concentrations of EO (4 MIC, 2 MIC, MIC, 1/2 MIC) and solvent (Control), and then adjust the concentration of bacteria again to 10^8^CFU/mL after anaerobic culture at 37°C and 180 rpm for 8 h. 70% ice ethanol was used as the positive control group. The staining was performed using the Live/Dead BacLight Bacterial Viability Kits (Invitrogen by Thermo Fisher Scientific, L7012), and the images were collected using the confocal microscope (Olympus FV3000).

### Scanning Electron Microscope and Transmission Electron Microscope

The effect of the EO on *C. acnes* morphology can be observed using SEM and TEM following the previously described method with modifications (Zhou et al., 2020). *C. acnes* with EO or solvent were anaerobically cultured with shaking at 180 rpm at 37°C for 8 h. The bacteria were collected by centrifugation at 4,500 rpm for 10 min at 4°C and washed three times in PBS. 2.5% glutaraldehyde solution was added and stored in a refrigerator at 4°C overnight. The bacteria were washed with 0.1 M phosphate buffer, centrifuged at 4°C 4,500 rpm for 10 min, and the bacteria were collected for standby.

(1) SEM: Bacteria were soaked in ethanol solutions of different concentrations (30, 50, 70, 90, and 100%) to remove water, then replaced with tert-butyl alcohol, freeze-dried and gilded, then observed and photographed by Thermal Field Emission Environmental SEM-Eds-EBSD (Quanta 400FEG).

(2) TEM: Fixed the sample with 1% Osmic acid solution for 1–2 h, rinsed the sample with 0.1 M phosphate buffer three times, then used the gradient dehydration of ethanol solution (30, 50, 70, 80, 90, 95, and 100%), and then treated 20 min with pure acetone. The sample was embedded with a mixture of the embedding agent and acetone overnight at 70°C, and then sliced with LEICA EM UC7 ultra-thin slicer. The slices were stained with lead citrate solution and uranyl acetate 50% ethanol saturated solution for 5–10 min, respectively. After drying, the slices could be observed under transmission electron microscopy (HITACHI H-7650).

### Metabonomics Studies

#### Sample Preparation

The GC-MS-based strategy was employed to analyze the metabolism profile of bacteria according to previous reports (Booth et al., 2015). 0.1% Tween 80 was also used as the solvent to enhance the EO solubility. Briefly, bacteria in the logarithmic phase were treated with EO (MIC) or solvent (Control) at 37°C for 8 h at 180 rpm, respectively. The bacteria were collected by centrifugation and washed three times in PBS. Then, 1 mL pre-cooled methanol was added to the quench, and the bacteria were lysed by a repeated freeze-thaw cycle followed by ultrasonication three times. The equal amount of the metabolites in the Control and MIC groups were mixed, respectively, with 8 μL ribitol (0.5 mg/mL) that was the internal standard and concentrated again. Then the derivatization reaction by using MSTFA was performed according to the previously reported method (Chen J. et al., 2020; Tang et al., 2021), followed detection within 48 h by GC-MS (Agilent 7890A GC equipped with Agilent 5975 CVL MSD Detector).

#### Gas Chromatography-Mass Spectrometry Detection Conditions

GC-MS detection was conducted according to the previously reported conditions with slight modifications (Chen J. et al., 2020; Tang et al., 2021). In brief, the injection volume was 1 μl in the split-less injection mode; Heating procedure: the initial temperature was 70°C, kept at the initial temperature for 3 min, then the temperature was heated to 285°C at 5°C/min, then to 310°C at 20°C/min, and kept at 310°C for 7 min; The ion source temperature was 230°C, and the four-stage rod temperature was 150°C. The carrier gas was He, and the flow rate was 1.0 mL/min.

#### Pre-processing of Metabolome Data

GC-MS data pre-processing was performed by Agilent Unknowns Analysis software version B.09.00. After peak identification and deconvolution, the metabolites were identified with NIST and Fiehn database. In addition, GC-MS data were subjected to the retention time correction and peak alignment. In addition, Metabolites signals were normalized using internal standards and quantile by R Statistical Computing Environment. These normalized data were used for subsequent analyses (The data of metabonomics included six biological replicates and two technical replicates for a total of 24 data).

#### Analysis of Metabolomics Data

Multivariate statistical analyses were carried out in R Statistical Computing Environment, including principal component analysis (PCA) and orthogonal partial least squares discriminant analysis (OPLS-DA). The metabolites of different groups can be screened by a combination of the variable importance projection (VIP) derived from the OPLS-DA model and the adjusted *p*-values (FDR, false discovery rate) of the Student’s *t*-test analysis. Finally, metabolites (VIP ≥ 1 and *p.adj* < 0.05) were selected as differential metabolites. In the differential metabolites, biomarkers were further selected according to the correlation and covariance information provided by S-plot. MBRole 2.0 pathway analysis of the differential metabolites was conducted using the KEGG database to determine that the metabolic pathways were considered significant with *p* < 0.05 (Lopez-Ibanez et al., 2016).

OmicStudio tools,^1^ Origin software (Origin Lab), and Adobe Illustrator were used to perform partial bioinformatics analysis and visualization.

### Detection of Enzyme Activity and Intracellular Adenosine Triphosphate Content

#### Enzyme Activity Assay

The bacteria concentration was adjusted to 10^9^CFU/mL as described before, and cultured with shaking in BHI medium with EO or solvent for 8 h as described in section “Bacteriostatic Curve.” After being washed twice in PBS, the bacteria were collected by centrifugation at 4,000 rpm at 4°C. According to the requirements of the kit, the extractant was added and extracted by the ultrasonic crusher for 10 min. The supernatant obtained by centrifugation was the extract, and BCA Protein Assay Kit detected the protein concentration. According to the instructions of the kits, detected the activities of the Pyruvate Decarboxylase (PC), Malate Dehydrogenase (MDH), Pyruvate kinase (PK), and Hexokinase (HK). Enzyme activity was expressed as units per milligram of protein. The detailed information of the enzymes activity determination process is depicted in Supplementary File 1.

#### Adenosine Triphosphate Content Assay

The bacterial liquid was collected and adjusted to 1.0 × 10^8^CFU/mL. The bacteria were treated, respectively, with EO at 1/2 MIC and MIC, or solvent (Control) as described in section “Bacteriostatic Curve,” and were cultivated at 37°C with shaking at 180 rpm for 8 h. Next, the bacteria were collected by centrifugation and washed twice in PBS. Liquid nitrogen was used to quench metabolism. The bacterial pellet was resuspended in PBS, and the bacterial liquid was 1.0 × 10^8^ CFU/mL. Then, the volume of BacTiter-Glo™ Reagent equal to the volume of the bacterial liquid in each well was added; after mixing and incubating for 5 min, the luminescence was recorded by an Infinite M1000 Proplate Reader (Tecan). In parallel, six adenosine triphosphate (ATP) concentration standards ranging from 2,500 pmol to 2,560 nmol were used to generate the ATP concentration standard curve by the BacTiter-Glo assay kit. Ultimately, the ATP concentration for each sample was calculated based on the standard curve.

### Statistical Analysis

Except for the analysis of metabolomics data, all statistical analyses were conducted in GraphPad Prism version 8.0.2. All the experiments were carried out in triplicate, with at least three biological replicates. The data were analyzed by one-way analysis of variance (ANOVA), represented as mean ± standard error of the mean (mean ± SEM). *p* ≤ 0.05 were regarded to be significant (^∗^*p* ≤ 0.05; ^∗∗^*p* ≤ 0.01; ^∗∗∗^*p* ≤ 0.001; ^∗∗∗∗^*p* ≤ 0.0001 vs. control group).

## Results

### Chemical Compositions of the Essential Oil

The major components of EO identified were listed in Supplementary Table 1. The top of 9 compounds accounted for 90.55%, which mainly included α-Citral (38.12%), β-Citral (32.97%), Limonene (9.72%), Linalool (2.45%), (R)-(+)-Citronellal (1.82%), Terpinen-4-ol (1.62%), α-Thujene (1.51%), Eucalyptol (1.21%), Caryophyllene (1.13%), in which the relative contents of α-citral and β-citral were higher, which was same as previously reported in the literature (Hu et al., 2019). The contents of Geraniol, β-Pinene, α-Terpineol, Sabinene, β-Myrcene, Camphene ranged from 0.53 to 0.94%. The others were less than 0.50%.

### Antibacterial Activity of Essential Oil Against *Cutibacterium acnes*

**FIGURE 1:**
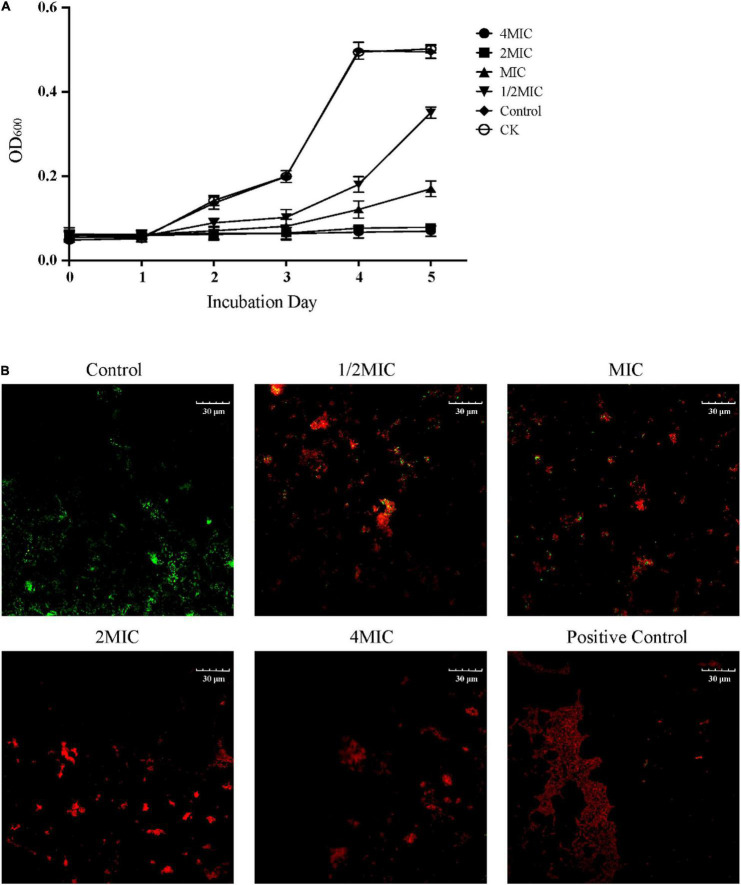
Inhibitory effect of EO on the growth of *Cutibacterium acnes*. **(A)** Effect of EO on the growth curve of *C. acnes* within 5 days. CK represented the blank control group and Control represented the solvent control group by 0.1% Tween 80. **(B)** Representative Live/Dead merged images (green and red) of *C. acnes* cultured for 8 h with solvent (Control) and different concentrations of EO (1/2 MIC, MIC, 2 MIC and 4 MIC). The positive control was treated with ethanol. Green fluorescence indicated living cells and red fluorescence indicated dead cells. The positive control was treated with ethanol.

The inhibitory effect of EO on the growth of *C. acnes* was evaluated by measuring the minimum inhibitory concentration (MIC) and the minimum bactericidal concentration (MBC). This result showed that EO could inhibit the growth rate of *C. acnes* in a dose dependent manner, which the MIC and the MBC values were 400 and 800 μg/mL, respectively (Supplementary Figure 1). As the results were shown in Figure 1A, there was no significant difference in bacterial growth between the solvent control group (Control) and the blank control group (CK), indicating that Tween 80 would not inhibit the growth of *C. acnes*. The growth of bacteria generally followed the model s-shaped growth curve, reached the logarithmic growth phase in 2 days and entered the platform phase 4 days later. The OD_600_ of *C. acnes* that co-cultured with EO at 1/2 MIC and MIC in each growth stage was significantly lower than that of the control group, and its growth was significantly inhibited. In the treatment with EO at MIC, *C. acnes* entered the logarithmic growth period at 4, 2 days later than the Control and CK. When treated with EO at 2 MIC and 4 MIC, *C. acnes* stopped growing.

### Bacterial Viability Test by Live/Dead Assay

The effect of EO on the activity of *C. acnes* was further observed by the Live/Dead BacLight Bacterial Viability assay, and each image represented the separate fluorescence channel (green and red) was shown in Supplementary Figure 2, the merge images was shown in Figure 1B. The cells produced green fluorescence but nearly undetectable red fluorescence in the control group, indicating the majority of cells were intact. After being cultured at 1/2 MIC and MIC in the medium with EO for 8 h, more spots with red fluorescence were observed. As the concentration of EO increased, the green fluorescence spots were gradually replaced by the red fluorescence spots, indicating that the number of bacterial death increased significantly and concentration-dependently. In the 2 MIC, and 4 MIC groups, the bacterial mortality rate was close to 100% (red fluorescence), which was similar to the positive control group.

### Scanning Electron Microscope and Transmission Electron Microscope

**FIGURE 2:**
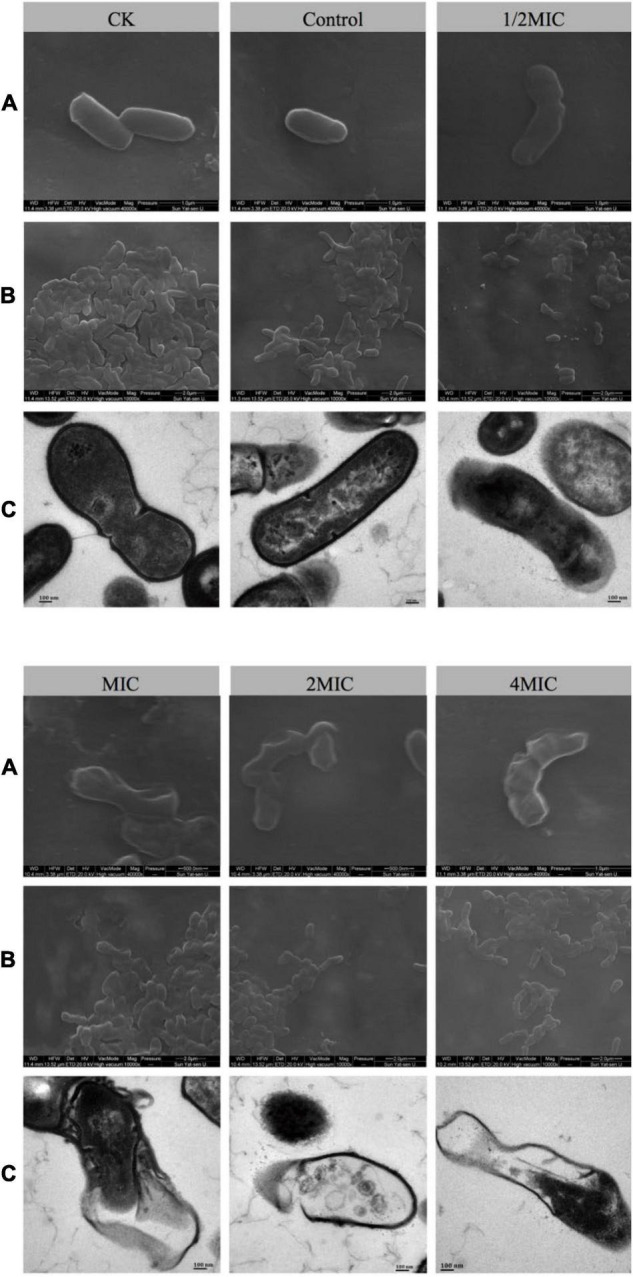
The effects of EO on the morphology of *C. acnes* cells were observed by scanning electron microscope (SEM) and transmission electron microscope (TEM), followed by normal bacteria (CK), solvent control bacteria (Control), and bacteria treated with different concentrations of EO (1/2 MIC, MIC, 2 MIC, and 4 MIC). **(A)** SEM image of *C. acnes* at 40,000× magnification. **(B)** SEM image of *C. acnes* at 10,000× magnification. **(C)** TEM image of *C. acnes* at 50,000× magnification.

In order to understand the intuitive effect of EO on the morphology of *C. acnes*, SEM and TEM techniques were used in this study (Wang et al., 2021). As shown in Figure 2, after 8 h of culture, the morphologies of the bacterium in the solvent control (Control) and the blank control group (CK) were complete, the surface of cells was intact, smooth, and bright, as well as the cells were full of cytoplasmic material. Treatment with 1/2 MIC for 8 h, most of the cell membranes were intact, but slightly concave and wrinkled. As for MIC, the bacterial cell membrane became blurred, the concave and fold of the cell membrane became more obvious, and the number of bacterial cell membrane damage increased. Besides, while treated with 2MIC or 4MIC, almost all of the cell membranes and cell walls were ruptured (Figure 2B). TEM can explore not only the morphology of bacterial cells more clearly, but also intracellular alteration of the bacteria. As shown in Figure 2C, the cells in the high concentration EO treatment group (4MIC and 2MIC) lysed, and the cytoplasmic material flowed out. The result showed that compared with the control group, the cell membrane and cell wall of the groups with EO were damaged, and the degree of damage increased with the increase of EO concentration.

### Metabonomics

#### Effect of Essential Oil on Metabolism of *Cutibacterium acnes*

**FIGURE 3:**
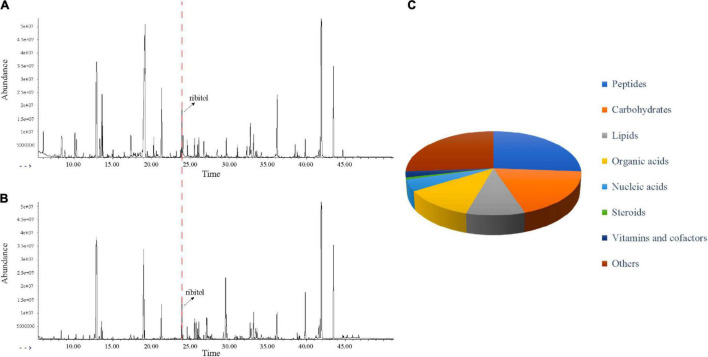
Metabolic profiling of *C. acnes* before and after EO treated. **(A)** Typical total ion chromatograms of the metabolic profiles of *C. acnes* with solvent (Control). **(B)** Typical total ion chromatograms of the metabolic profiles of *C. acnes* with EO (MIC). **(A,B)** Peaks were aligned and internal standard (ribitol) was highlighted. **(C)** KEGG analysis showed different types of metabolites identified.

In this study, GC-MS was used to analyze the effect of EO on bacterial metabolism. According to the result in Figures 3A,B, there were significant differences in the abundance of substances with the same retention time between the MIC and Control groups. As shown in Figure 3C, after GC-MS detection and data preprocessing of 24 groups of samples, a total of 133 metabolites were obtained, including peptides (26.3%), carbohydrates (18.8%), lipids (9.8%), organic acids (12.0%), nucleic acids (4.5%), vitamins and cofactors (2.3%), steroids (0.8%), and others (25.6%).

#### Analysis of Differential Metabolites

**FIGURE 4:**
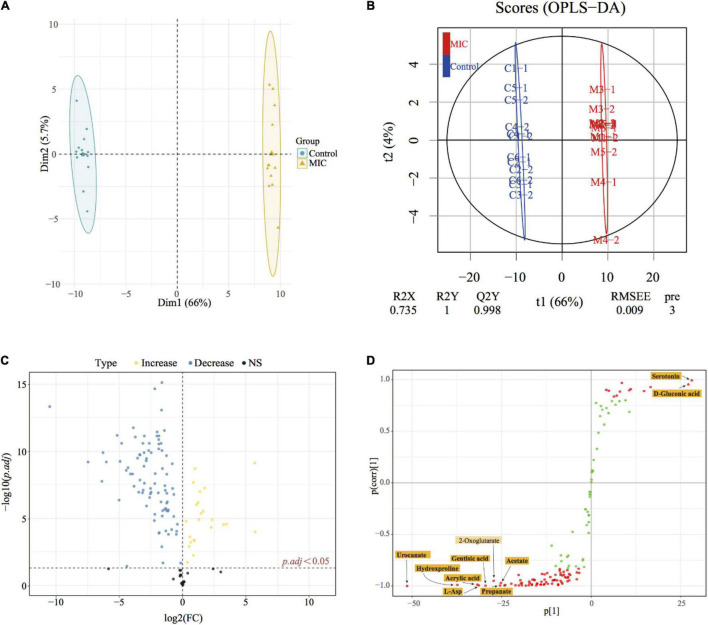
Metabonomics statistical analysis. **(A)** PCA score plot; **(B)** OPLS-DA score plot (*R*^2^X = 0.735, *R*^2^Y = 1 *Q*^2^Y = 0.998). C#-# and M#-# presented the sample name of the control group and MIC group, respectively. **(C)** Volcano plot; yellow dots represented the increase in metabolite abundance, and blue dots represented the decrease in metabolite abundance (vs. Control). *p.adj* represented adjusted *p*-values using FDR. **(D)** S-plots from the OPLS-DA model, the abscissa indicated the covariance, and the ordinate indicated correlation.

**FIGURE 5:**
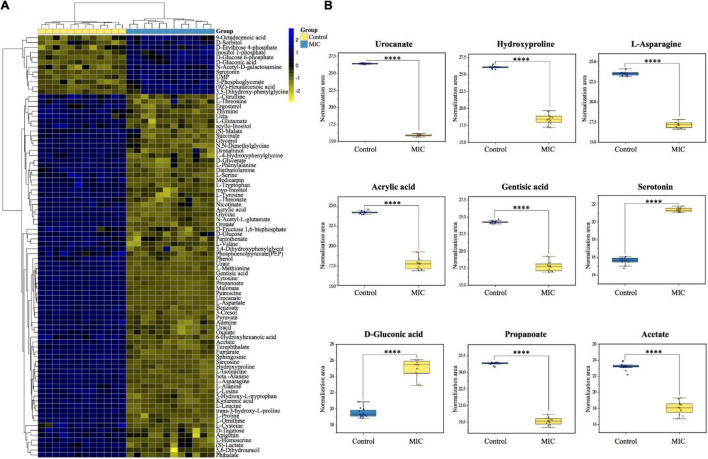
Differential metabolite heat map and biomarker identification. **(A)** The heatmap was constructed based on the differential metabolites in MIC group and Control group (*n* = 12). The color key indicated metabolite abundance (blue: upregulation; yellow: downregulation). Rows: metabolites; Columns: samples. Control and MIC group were colored yellow and cyan, respectively. **(B)** The boxplots of biomarkers to show the difference between MIC group and Control group.

Multivariate statistical analysis and univariate statistical analysis were combined to study the metabolic differences between the EO-treated group (MIC) and the control group. The metabolites were analyzed by PCA, on the score plot (Figure 4A), the natural clustering trend among metabolites was observed, and it was found that MIC group and Control group could be obviously separated. The supervised OPLS-DA model was established to compare the metabolic changes between MIC and Control groups. The *Q*^2^ value of the model was 0.998, indicating that it had high validated predictability and could be used for further screening of differential metabolites (Figure 4B). In the S-plot of OPLS-DA (Figure 4D), each spot was representative of a compound, and the further away from the origin, the more significant its contribution to clustering of the two groups. The contribution was represented by Variable Importance in Project (VIP) value, and the red spots in the figure indicated the compound with VIP ≥ 1. 110 metabolites were selected by Student’s *t*-test calculated significant differences between the two groups (*p.adj* < 0.05), and the Fold Change was calculated to quantify the degree of up/downregulation of metabolites, as shown in Figure 4C. Only if the VIP was above 1 and *p.adj* was below 0.05, the metabolites could be considered as differential metabolites. A total of 86 endogenous metabolites were selected as differential metabolites before and after the administration of EO and classified (Supplementary Figure 3). The heat map of differential metabolites showed that the abundance of *C. acnes* metabolites changed significantly after EO administration, with 74 metabolites decreased and 12 increased (Figure 5A). (Other detailed metabolic information, including VIP values in multivariate statistical analysis, *p*. *adj* and Fold Change in univariate statistical analysis, were listed in Supplementary File 3.)

#### Discovery of Biomarkers

Biomarker discovery is the critical step for metabolomics studies. According to the 86 differential metabolites that had been selected (Figure 5A), combined with 10 candidate biomarkers obtained by S-plot (highlighted in Figure 4D), it was found that 9 metabolites (Figure 5B) can be used as biomarkers, namely urocanate, hydroxyproline, L-asparagine, acrylic acid, gentisic acid, serotonin, D-gluconic acid, propanoate, and acetate, which belonged to peptides, organic acids, carbohydrates and other kinds, respectively. However, the 2-oxoglutarate was excluded because the *p.adj* was larger than 0.05.

#### Enrichment of Metabolic Pathway

**FIGURE 6:**
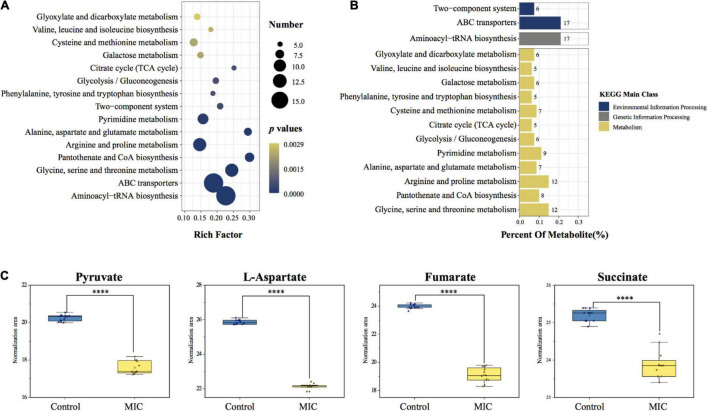
Metabolic pathway enrichment analysis and critical metabolites involved in the pathway. **(A)** KEGG enrichment analysis bubble chart of metabolic pathways. The x-axis represented the rich factor (the number of differential metabolites enriched in the pathway). The y-axis represented the name of KEGG pathways. The size of the bubble represented the number of the differential metabolites, and the color represented *p*-values. **(B)** KEGG classification of enriched pathways. The number next to the bar represented the number of differential metabolites involved in the pathway. (Percent Of Metabolite: the proportion of differential metabolites involved in this pathway to the total differential metabolites.) **(C)** Changes in the abundance of critical metabolites before and after administration.

A total of 34 metabolic pathways (*p.adj* < 0.05) were obtained by metabolic pathway enrichment analysis of 86 differential metabolites (Supplementary File 4). According to the order of *p.adj* from small to large (the smaller the *p.adj*, the more significant the impact on the metabolic pathway), the top 15 of them were shown in Figure 6A. 34 metabolic pathways were subdivided into the three kinds of KEGG main class, of which metabolism-related pathways accounted for 88.2%, including carbohydrate metabolism, amino acid metabolism and metabolism of cofactors and vitamins; In addition, 8.8% of environmental information processing-related pathways included membrane transport and signal transduction; and 3.0% of genetic information processing-related pathways included translation (Supplementary File 4). KEGG classification of the top 15 metabolic pathways was shown in Figure 6B. Among the differential metabolites involved in the enrichment pathway, we found that pyruvate, L-aspartate, fumarate, and succinate were the critical intermediates of the metabolic pathway. Their abundance changes before and after EO administration were shown in Figure 6C.

### Enzyme and Adenosine Triphosphate Content Test

**FIGURE 7:**
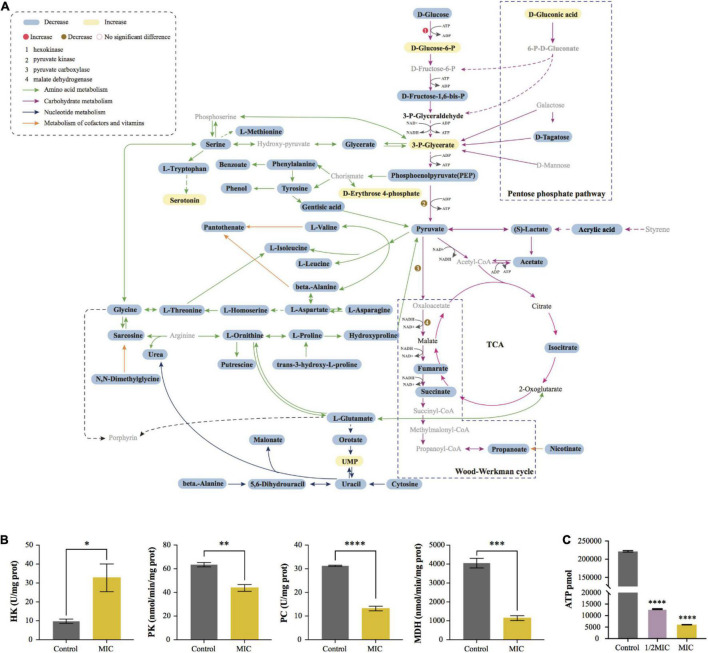
Visualization of metabolic pathway and changes of key enzyme activity and ATP content before and after EO treatment. **(A)** Summary graph of metabolic pathways in MIC group (vs. Control). The black fonts indicated the detected metabolites (blue boxes were significantly down-regulated differential metabolites, yellow boxes were significantly up-regulated metabolites, and the rest are not differential metabolites), while the gray fonts indicated undetected metabolites. The activities of Hexokinase (HK), Pyruvate kinase (PK), Pyruvate Decarboxylase (PC) and Malate Dehydrogenase (MDH) are shown in **(B)**. **(C)** Effect of EO on ATP synthesis in *C. acnes* cells.

Based on the result from enrichment analysis, the metabolic pathways of *C. acnes* under the influence of EO were plotted (Figure 7A). In order to further verify the effect of EO on *C. acnes* metabolism, the activities of PC and MDH, enzymes related to the Wood-Werkman cycle, as well as the activity of HK and PK, the key enzyme in glycolysis were measured, and the change of intracellular ATP content was determined. The results were shown in Figures 7B,C. Compared with the control group, the activity of HK increased significantly, and the activities of PC, MDH, and PK decreased significantly after MIC treatment for 8 h, as well as the intracellular ATP content. After treating with EO, the activities of PC, MDH, and PK in the MIC group were lowered by approximately 57.85, 71.78, and 30.91%, respectively. However, the activity of HK was increased by 70.13% (Figure 7B). Additionally, the ATP content in the 1/2 MIC and MIC groups was downregulated 94.21 and 97.27% compared to the control group (Figure 7C).

## Discussion

In this study, we found that *Litsea cubeba* essential oil (EO) had sound antibacterial and bactericidal effects on *C. acnes* (ATCC6919), and the inhibitory effect on bacteria increased with the increase of the concentration of EO. This discovery not only expanded the use of EO, but also took the first step for us to find new drugs for the treatment of *C. acnes*-related diseases.

It is well known that the integrity of bacterial cell walls and cell membranes was the key factor for bacterial survival (Call and Klaenhammer, 2013). The bacterial cells stained by Live/Dead staining assay showed the cell viability of five groups of EO with different concentrations after co-culture with *C. acnes* for 8 h (Figure 1B and Supplementary Figure 2). The number of dead cells (red) in the administration groups increased significantly and showed concentration dependence, which was consistent with the antibacterial activity test results (Figure 1A). It also showed that the integrity of *C. acnes* cell membrane is damaged, which was more obvious in the images taken by SEM and TEM (Figure 2). The disrupted bacterial cellular integrity, as well as the emerging of cell debris, confirmed that the EO could alter the bacterial morphology.

Metabonomics is a qualitative and quantitative method of small molecular metabolites in the biological system utilizing LC-MS or GC-MS or NMR (Hu and Xu, 2014; Pang et al., 2019), which can understand the life activities of organisms comprehensively and systematically. This method has been used to evaluate the mechanism of bacterial metabolism and bacteriostatic mechanism (Wong et al., 2018). In this study, non-target metabonomics based on GC-MS was used to explore the metabolic changes of *C. acnes* before and after the administration of EO, which has hardly ever been done in prior studies. Furthermore, the results from the perspective of metabolomics demonstrated that metabolic pathways of *C. acnes*, such as glycolysis, Wood-Werkman cycle and pentose phosphate pathway (Figures 6A,B), were similar to the *Propionibacterium* spp. though *C. acnes* has now been reclassified as *Cutibacterium* spp. (Scholz and Kilian, 2016), which was consistent with findings in most previous studies by other omics methods (McLaughlin et al., 2019). Therefore, the metabonomics study of other *Propionibacterium* spp. can provide references for us.

In this study, we identified a number of significantly altered metabolites (86 differential metabolites) after the administration of EO to *C. acnes*, in which the 9 metabolites was selected as biomarkers (the biomarker pattern is not unique to the antibacterial mechanism of EO) (Figure 5). Based on the above metabolomic analysis and previous studies on *Propionibacterium* spp., *C. acnes* showed a complex biological network to cope with EO-treated, which may be related to the carbohydrate metabolism, energy metabolism, amino acid metabolism, as well as cell wall and cell membrane synthesis (Figure 7A and Supplementary File 4).

### Carbohydrate Metabolism

The glycolysis and propanoate production pathways are the central pathways of carbohydrate metabolism in *C. acnes*. Hexokinase (HK) and pyruvate kinase (PK) are rate-limiting enzymes in the glycolysis pathway. HK is the first enzyme of the glycolysis to catalyze the phosphorylation of glucose to glucose-6-phosphate, and pyruvate, the end product of the glycolysis pathway, is produced by PEP under PK. In our study, we observed a significant increase in the catalytic activity of HK, and the phenomenon may be caused by the defense response of *C. acnes* under environmental stress, which can avoid the damage of EO on bacterial metabolism by increasing glucose uptake, according to the literature investigation (Figures 6C, 7A,B; Wang et al., 2018). Pyruvate is the critical hub of the pathways in carbohydrate and amino acid metabolism, in the Propionibacterium genus, the synthesis of propanoate through the Wood-Werkman cycle is one of the essential pathways of pyruvate metabolism (McCubbin et al., 2020). Pyruvate forms oxaloacetate under pyruvate carboxylase (PC), which starts the first step of the propanoate synthesis. Malate dehydrogenase (MDH) converts oxaloacetate to malate, which is then converted to fumarate by succinate reductase, and fumarate yields succinyl-CoA and finally to propanoate. Our results demonstrated that, during this process, the abundance of 3 critical intermediates (pyruvate, fumarate, and succinate) of the metabolic pathway, and propanoate, the end product of the Wood-Werkman cycle after EO-treated decreased significantly (Figures 5B, 6C), indicating that the Wood-Werkman cycle was inhibited. The activities of MDH and PC downregulated, which also confirmed this result (Figure 7B).

### Energy Metabolism

ATP is the direct energy source for bacteria to maintain their life and normal physiological activities (Venkateswaran et al., 2003). Unlike other common aerobic bacteria, in *C. acnes*, the Wood-Werkman cycle, which can be coupled with an anaerobic electron transport chain (anaerobic respiration), is the primary energy supply pathway. NADH can provide H^+^, transfer H^+^ to fumarate through a series of enzymes to drive the proton pump to synthesize ATP (de Vries et al., 1973). Moreover, *C. acnes* also utilize substrate-level phosphorylation to produce ATP, such as glycolysis and pyruvate to acetate. Although acetate is a by-product of the metabolism, it is vital to *C. acnes* (McCubbin et al., 2020). Our result suggested that, as a biomarker, the abundance of acetate decreased significantly after EO treatment, indicating that the pathway of pyruvate to acetate was inhibited. Similarly, we found that other metabolisms related to ATP synthesis, including the Wood-Werkman cycle and glycolysis, were also disturbed, suggesting that EO inhibited the ATP synthesis of bacteria, leading to the inhibition of their growth. It was consistent with the result of ATP content in bacterial cells detected (Figure 7C).

### Amino Acid Metabolism

The result indicated that EO also had a significant effect on the metabolism of amino acids in *C. acnes* because the abundance of almost all amino acids in bacterial cells was significantly reduced after EO-treated (Figure 7A). Previous research has shown that L-Aspartate, the main precursor of amino acid synthesis in microorganisms, is key to amino acid metabolism and bacterial growth (Qiao et al., 2018). The results (Figures 6C, 7A) demonstrated that, as one of the four critical intermediates of the metabolic pathway, the inhibition of L-aspartate synthesis may cause the inhibition of the whole amino acid metabolism. Amino acid metabolism is a crucial metabolic pathway for bacterial cells to grow and maintain normal life activities (Afzal et al., 2016). It involves many aspects, such as protein synthesis, biological enzyme synthesis and regulation, gene expression, osmotic regulation, and so on (Wu, 2009). Therefore, disruption of amino acid metabolism will lead to abnormal physiological activities of bacteria. Apart from the above, amino acid metabolism also involves the synthesis of virulence factors in bacteria. *C. acnes* can secrete porphyrin, hyaluronidase, lipase, and others to cause cell inflammation, which is closely related to the pathogenesis of *acne* (De Canha et al., 2019; Spittaels et al., 2021). The disorder of amino acid metabolism may inhibit the ability of bacteria to secrete virulence factors and reduce their pathogenicity. However, this hypothesis needs to be tested further.

### Cell Wall and Cell Membrane Synthesis

The cell wall of *C. acnes* is mainly composed of peptidoglycan, fatty acid and polysaccharide. Peptidoglycan is a disaccharide peptide consisting of sugar and amino acid, crossing links with each other to form a polymer network outside the cell membrane, which exists in almost all bacteria (Prasad et al., 2019). The results showed that the abundance of glucose decreased significantly, and the amino acid metabolism was disturbed (the abundance of alanine, glutamate, glycine and other amino acids decreased significantly). As the important precursors of synthetic peptidoglycan according to relevant literature (Johnson and Cummins, 1972), the decrease of their abundance indicated that the bacterial cell wall might be destroyed (Figure 7A and Supplementary File 3). It is worth noting that in *C. acnes* treated with EO, the abundance of these two unsaturated fatty acids 9-Octadecenoic acid and (9Z)-Hexadecenoic acid, main components of the cell wall and cell membrane, increased significantly, indicating that the synthesis and repair of the cell wall and cell membrane were strongly up-regulated (Supplementary File 3). It has been shown in the literature that microorganisms would protect themselves by the increase of unsaturated fatty acids to avoid damage caused by the administration, which was a stress response (Knoll et al., 2021). Therefore, it could be suggested that the damage of the EO to the cell wall and the cell membrane of the *C. acnes* may be indirectly caused by the influence of metabolism.

## Conclusion

To sum up, we studied the antibacterial activity of *Litsea Cubeba* essential oil (EO) against *Cutibacterium acnes* (*C. acnes*) and established a metabonomics method based on GC-MS to investigate the related metabolic changes and the bacteriostatic mechanism of *C. acnes* after administration. Our results demonstrated that EO could inhibit the growth of *C. acnes* and destroy bacterial cell structure. Metabolomics analysis showed that EO treatment could significantly affect 34 metabolic pathways of *C. acnes*, including carbohydrate metabolism, energy metabolism, amino acid metabolism, as well as cell wall and cell membrane synthesis, which finally disturbs normal bacterial metabolism. Overall, this discovery is expected to deepen the understanding of the activity of EO, widen its application range and improve its commercial value.

## Data Availability Statement

The original contributions presented in the study are included in the article/Supplementary Material, further inquiries can be directed to the corresponding author/s.

## Author Contributions

JC conducted the experiments. JC and JZ performed data analysis and wrote the manuscript. JC, JZ, LZ, CQ, and HT contributed to discussing the results and critical review of the manuscript. ZZ reviewed, edited the manuscript, and acquired funding. LJ performed data interpretation, reviewed, and edited the manuscript. DY performed data interpretation, reviewed and edited the manuscript, provided supervision and project administration, and acquired funding. All authors made significant contributions to this article and participated actively in the conception and design of the experiments, reading, and approving the final manuscript.

## Conflict of Interest

The authors declare that the research was conducted in the absence of any commercial or financial relationships that could be construed as a potential conflict of interest.

## Publisher’s Note

All claims expressed in this article are solely those of the authors and do not necessarily represent those of their affiliated organizations, or those of the publisher, the editors and the reviewers. Any product that may be evaluated in this article, or claim that may be made by its manufacturer, is not guaranteed or endorsed by the publisher.

## Footnotes

1
https://www.omicstudio.cn/tool
