# Supplementary material for: Antibacterial Activity of the Essential Oil From Litsea cubeba Against Cutibacterium acnes and the Investigations of Its Potential Mechanism by Gas Chromatography-Mass Spectrometry Metabolomics
Source: Front Microbiol. 2022 Mar 2;13:823845. doi: 10.3389/fmicb.2022.823845 (PMC8924494; doi:10.3389/fmicb.2022.823845)
Supplement: Supplementary file 1 [file Data_Sheet_1.DOCX]

Supplementary Material

# Supplementary Method

**GC-MS conditions for identification of chemical constituents of EO:** A DB-5H column (30.0 m ×0.25 mm ×0.25 μm) was adopted in this system. Operating conditions in the GC-MS detection were as follows. Injection volume: 1 μL. The oven temperature was programmed at 40 °C for 5 min, increased to 220 °C at 5 °C/min and held for 15 min. The energy was 70 eV in the electron impact mode. The mass spectrometry data: 35–300 m/z full-scan.

**The definition and calculation of metabolism-related enzyme activities:**

(a) Pyruvate Decarboxylase (PC) activity: one enzymatic unit was defined as the quantity of per milligram of enzyme protein required to consume 1 nmol of NADH per minute.

PC (U/mg prot)= 964.2×(ΔA_Sample_-ΔA_Blank_)÷Cpr (PC)

(b) Malate Dehydrogenase (MDH) activity: one enzymatic unit was defined as the quantity of per milligram of enzyme protein required to consume 1 nmol of NADH per minute.

MDH(U/mg prot)= 12860×(ΔA_Sample_-ΔA_Blank_) ÷Cpr (MDH)

(c) Pyruvate kinase (PK) activity: one enzymatic unit was defined as the quantity of per milligram of enzyme protein required to consume 1 nmol of NADH per minute.

PK(U/mg prot)= 2680×(ΔA_Sample_-ΔA_Blank_)÷Cpr (PK)

(d) Hexokinase (HK) activity: one enzymatic unit was defined as the quantity of per milligram of enzyme protein required to produce 1 nmol of NADPH per minute.

HK(U/mg prot)= 1071.7×(ΔA_Sample_-ΔA_Blank_)÷Cpr (HK)

Blank in the above-described experiments was determined using distilled water in place of the sample.
