# Supplementary material for: Antibacterial Activity of the Essential Oil From Litsea cubeba Against Cutibacterium acnes and the Investigations of Its Potential Mechanism by Gas Chromatography-Mass Spectrometry Metabolomics
Source: Front Microbiol. 2022 Mar 2;13:823845. doi: 10.3389/fmicb.2022.823845 (PMC8924494; doi:10.3389/fmicb.2022.823845)
Supplement: Supplementary file 2 [file Data_Sheet_2.DOCX]

Supplementary Material

# Supplementary Table

**Table S1: Chemical composition of the EO.**

| NO. | Compound | Percentage (%) | RI^a^ | RI^b^ |
| --- | --- | --- | --- | --- |
| 1 | α-citral | 38.12 | 1267.71 | 1270 |
| 2 | β-citral | 32.97 | 1238.08 | 1240 |
| 3 | Limonene | 9.72 | 1028.77 | 1018 |
| 4 | Linalool | 2.45 | 1097.37 | 1099 |
| 5 | (R)-(+)-Citronellal | 1.82 | 1150.39 | 1152 |
| 6 | Terpinen-4-ol | 1.62 | 1180.35 | 1177 |
| 7 | α-thujene | 1.51 | 932.69 | 929 |
| 8 | Eucalyptol | 1.21 | 1031.58 | 1032 |
| 9 | Caryophyllene | 1.13 | 1423.55 | 1419 |
| 10 | Geraniol | 0.94 | 1247.96 | 1255 |
| 11 | β-Pinene | 0.83 | 946.73 | 943 |
| 12 | α-Terpineol | 0.83 | 1194.10 | 1189 |
| 13 | Sabinene | 0.71 | 972.07 | 974 |
| 14 | β-Myrcene | 0.66 | 988.29 | 991 |
| 15 | Camphene | 0.53 | 949.28 | 952 |
| 16 | Others | 4.94 | -- | -- |

RI^a^: the retention indices were calculated by the homologous series of n-alkanes C7-C40 on HP-5MS column. RI^b^: Retention indices in the NIST Mass Spectral Library.

# Supplementary Figure


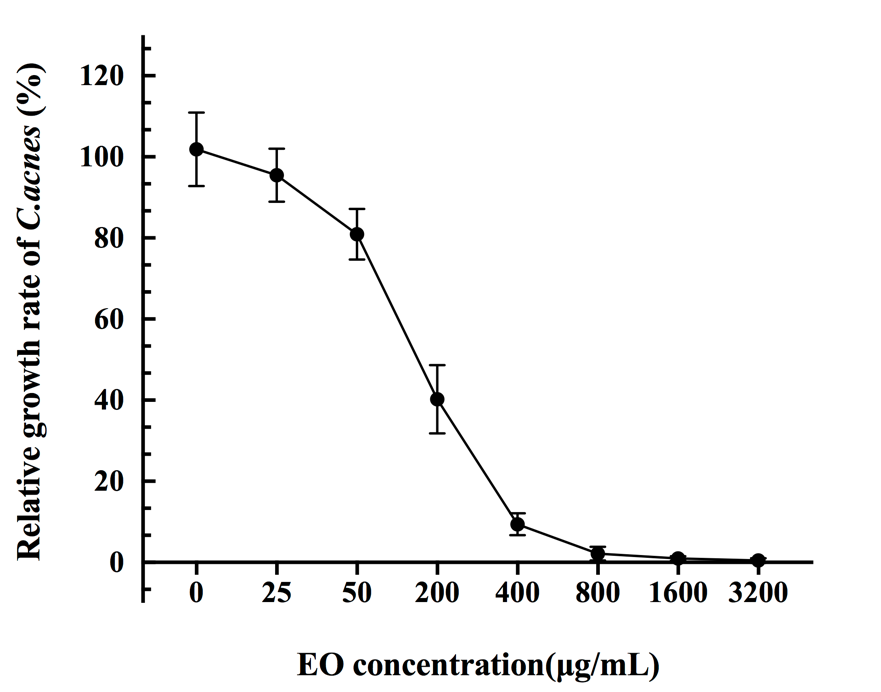


**Figure S1: Relative growth rate of *C.acnes* co-cultured with different EO concentrations.** The relative growth rate was expressed as a percentage of the OD_600_ value of the blank control group (CK) which was assigned a value of 100%. The vehicle control was represented as 0 μg/mL concentration of EO. Data were expressed as mean ± SEM.


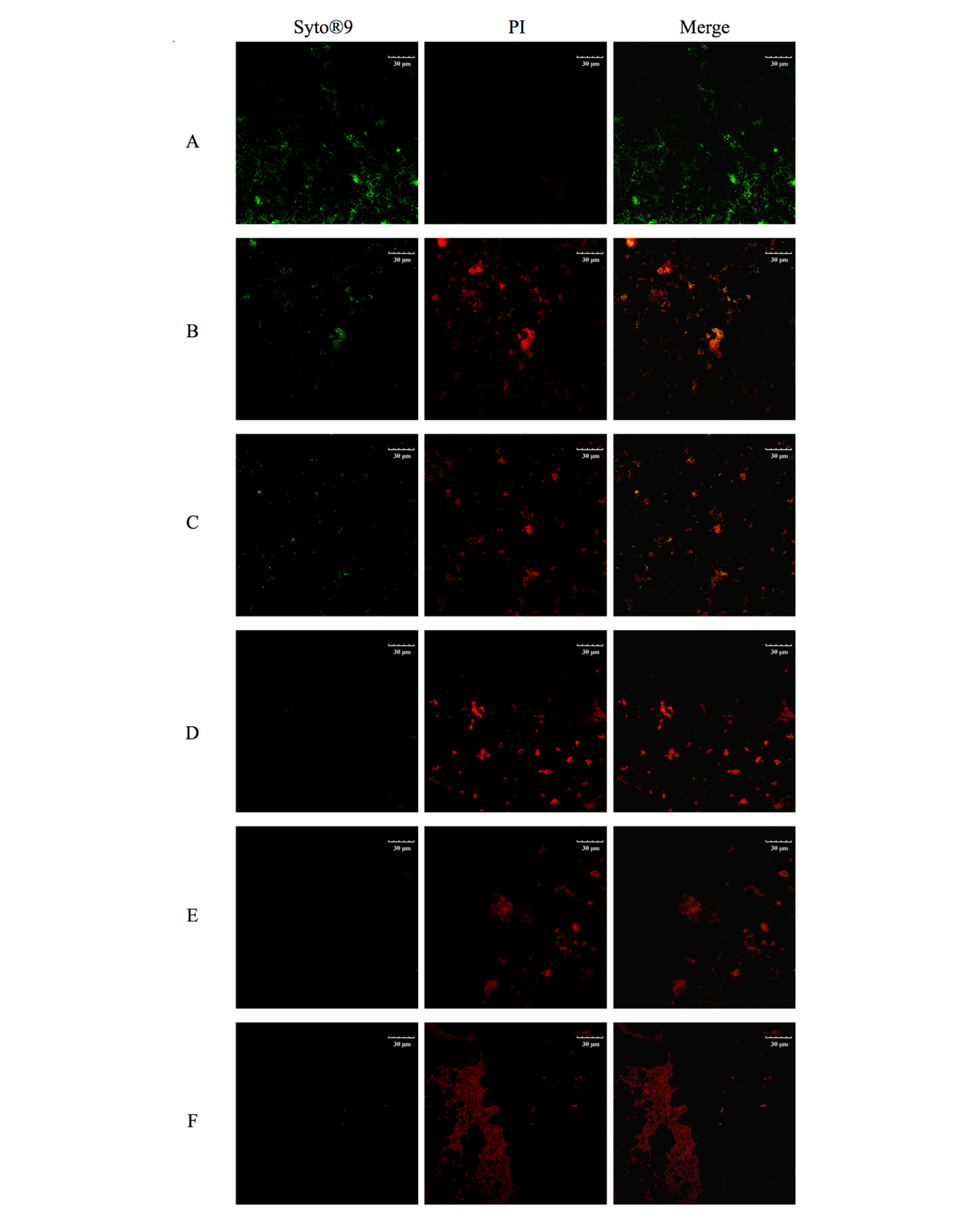


**Figure S2: Fluorescence of *C.acnes* cells stained with Live/Dead BacLight^TM^ Bacterial Viability kit.** (A) Control, (B) 1/2 MIC, (C) MIC, (D) 2 MIC, (E) 4MIC, (F) Positive Control. Shown were separate monochrome images of the green fluorescence channel (Syto®9), the red fluorescence channel (PI) and merged color images from all channels.


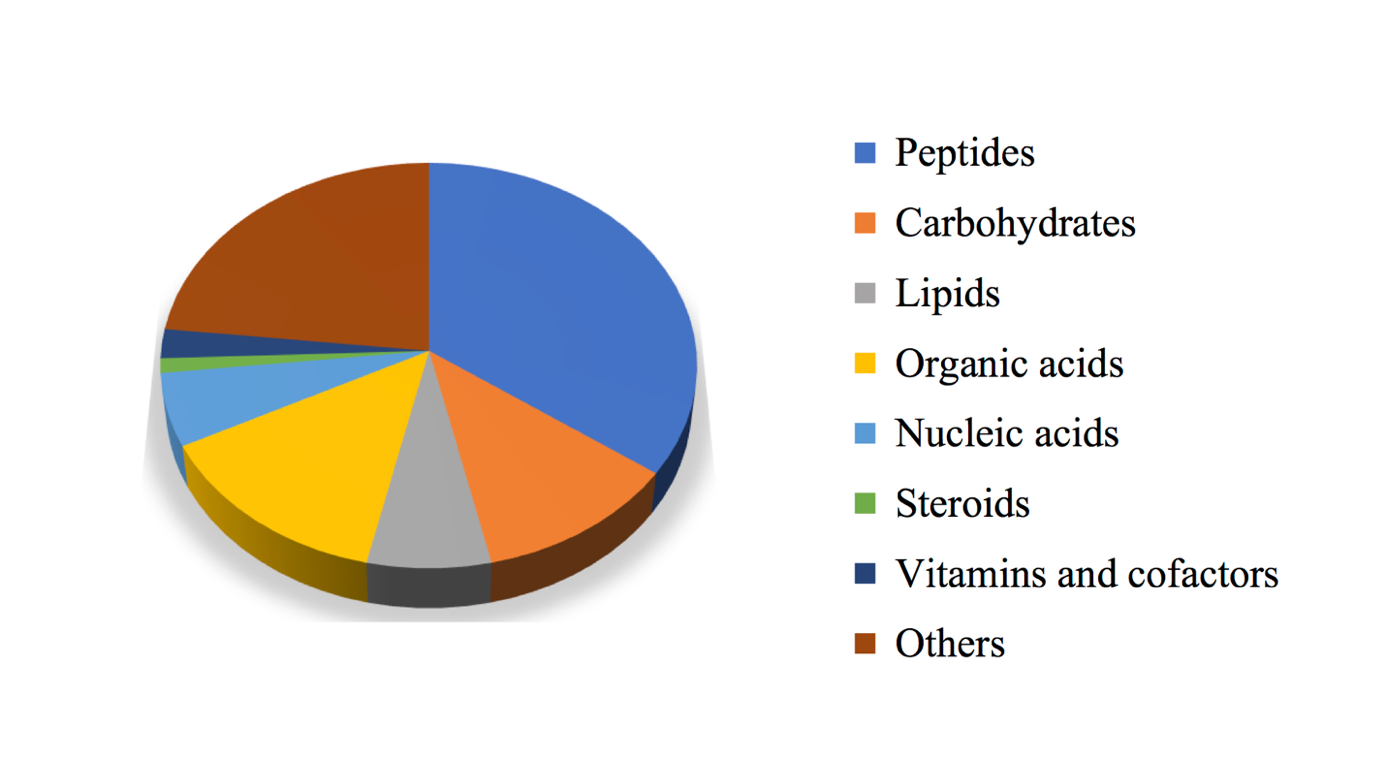


**Figure S3: Differential metabolites identified between MIC and Control groups.** Peptides accounted for 34.9 %, Carbohydrates 11.6 %, Lipids 7.0 %, Organic acids 14.0 %, Nucleic acids 5.8%, Steroids 1.2 %, Vitamins and cofactors 2.3 %, Others 23.3 %.
